# Biodegradable, Flame-Retardant, and Bio-Based Rigid Polyurethane/Polyisocyanurate Foams for Thermal Insulation Application

**DOI:** 10.3390/polym11111816

**Published:** 2019-11-05

**Authors:** Marcin Borowicz, Joanna Paciorek-Sadowska, Jacek Lubczak, Bogusław Czupryński

**Affiliations:** 1 Department of Chemistry and Technology of Polyurethanes, Institute of Materials Engineering, Kazimierz Wielki University, J. K. Chodkiewicza Street 30, 85-064 Bydgoszcz, Poland; 2 Department of Organic Chemistry, Rzeszów University of Technology, Al. Powstańców Warszawy 6, 35-959 Rzeszów, Poland; jml@prz.edu.pl

**Keywords:** mustard seed oil, rigid polyurethane/polyisocyanurate foam, bio-polyol, thermal insulation

## Abstract

This article raised the issue of studies on the use of new bio-polyol based on white mustard seed oil and 2,2’-thiodiethanol (3-thiapentane-1,5-diol) for the synthesis of rigid polyurethane/polyisocyanurate (RPU/PIR) foams. For this purpose, new formulations of polyurethane materials were prepared. Formulations contained bio-polyol content from 0 to 0.4 chemical equivalents of hydroxyl groups. An industrial flame retardant, tri(2-chloro-1-methylethyl) phosphate (Antiblaze TCMP), was added to half of the formulations. Basic foaming process parameters and functional properties, such as apparent density, compressive strength, brittleness, absorbability and water absorption, aging resistance, thermal conductivity coefficient λ, structure of materials, and flammability were examined. The susceptibility of the foams to biodegradation in soil was also examined. The increase in the bio-polyol content caused a slight increase in processing times. Also, it was noted that the use of bio-polyol had a positive effect on the functional properties of obtained RPU/PIR foams. Foams modified by bio-polyol based on mustard seed oil showed lower apparent density, brittleness, compressive strength, and absorbability and water absorption, as well as thermal conductivity, compared to the reference (unmodified) foams. Furthermore, the obtained materials were more resistant to aging and more susceptible to biodegradation.

## 1. Introduction

The economic doctrine of sustainable development keeps the balance between the economy, care for the environment, and quality of life. The fundamental principle of sustainable development is achieving the status that all basic human needs are met while maintaining the integrity of the Earth’s ecosystem [1,2]. When it comes to polyurethane (PU) plastics, sustainable development mostly concerns two scopes. The first is the replacement of components derived from nonrenewable sources with their renewable counterparts. This main aim is a limiting of the extraction and processing of fossil raw materials and the reduction of the environmental degradation caused by this process. In turn, the second aim refers to the reduction of nondegradable waste emissions to the environment. It is possible, e.g., by introducing formulations into the ingredients which degrade in biotic and abiotic conditions [3]. This approach prevents depositing post-consumer waste in landfills or their thermal utilization, which is accompanied by the emission of environmentally toxic gases, such as hydrogen cyanide [4].

The most popular method of implementing the concept of sustainable development in the polyurethane industry is the use of components based on natural origin raw materials (e.g., bio-polyols or bio-fillers) for their production [5,6,7,8,9].

Research on the use of soybean oil-based bio-polyols was conducted by Miao et al. Authors used soy-polyols for the production of different kinds of polyurethane materials. On the basis of this bio-polyol, PU elastomer composites were obtained and characterized by very good mechanical properties and high cellular biocompatibility. The last parameter had particular importance in biomedical applications, e.g., in the implant production. The second bio-polyol was used to obtain foams with the so-called “shape memory”. The research results proved that the use of soybean-based bio-polyols improved the properties and opened up new direction for applications for this material [10,11,12]. Veronese et al. used a mixture of petrochemicals and soy-based polyols for the production of rigid polyurethane foams. The obtained materials had better mechanical properties than their counterparts, which were obtained from petrochemical raw materials [13]. Dhaliwal et al. obtained polyurethane foams containing a polyol based on soybean oil. It was noted that the soy-based polyol was a valuable raw material. It enabled the production of PU foams with apparent density and thermal insulation properties comparable to the control sample and much better dimensional stability [14]. They also tested polyurethane foam containing a soybean oil-based polyol for insulation and structural applications. It was found that PU foam obtained using soy-based polyol can replace conventional petroleum-based PU foam for thermal insulation. A number of formulations were developed with varying amounts of blowing agent and catalyst to optimize the mechanical and thermal properties of soy-based PU foams. Based on the obtained test results, it was noted that the foam modified with vegetable polyol showed more favorable strength characteristics and better thermal properties in comparison to foams based on petrochemical raw materials [15].

Rapeseed oil-based bio-polyol was used to obtain all types of polyurethane foams, as well as elastomers, adhesives, and coatings [16]. Kurańska et al. did research on the replacement of petrochemical polyol for a bio-polyol based on rapeseed oil. The studies regarded the production of rigid polyurethane and polyurethane–polyisocyanurate foams. The obtained materials had similar properties (such as thermal conductivity coefficient, water absorption, closed cell contents) as unmodified foams [8,16]. Malewska et al. tested the effect of various rapeseed oil-based polyols concerning the properties of flexible polyurethane foams. The raw materials differed from each other due to the chemical structure and the hydroxyl number because various glycols were used to open the oxirane rings. Research showed that 20% of rapeseed-based bio-polyols had a positive effect on functional properties, such as hardness and comfort factor [17,18].

Palm oil-based polyol is a crucial bio-polyol raw material for the production of polyurethanes. Research on its application is conducted mainly in Asia—its largest producer (about 90% of world production) [7]. Badri et al. obtained a bio-polyol based on the transesterification process of glycerides contained in vegetable oils with diethanolamine. It was used, inter alia, for the synthesis of polyurethane foams. Materials based on it were characterized by very good thermal insulation properties, strong adhesion to various surfaces, low water absorption, and high resistance to many aggressive environmental factors, e.g., acids, alkalis, sea salt, or organic solvents [19,20]. The same bio-polyol was used by Su’ait et al. to produce a solid electrolyte (PU–LiI) photovoltaic cell. The chemical reactions which occur in the electrolyte–electrode system are the source of the electric current generated by the solar panel. Studies have shown chemical interactions between segmented polyurethane based on plant origin raw material and lithium ion. The obtained bio-composite had high electrical conductivity, which was a noteworthy alternative to conventional electrolytic systems [21]. Septevani et al. conducted research on the influence of palm oil-based polyesterol on the properties of rigid polyurethane foams. Bio-polyol was used at various weight ratios in a mixture with petrochemical polyetherol. Composites containing 30% by weight of palm-based polyesterol in the mixture of polyol components were characterized by better mechanical properties [5]. Adnan et al. used bio-polyol based on palm-olein for the synthesis of high-resilient (HR) polyurethane foams. The conducted research showed that the implementation of vegetable-based polyol up to 25 wt.% did not have any influence on properties of the obtained materials [22].

The main aim of this research was to examine the effect of bio-polyol based on mustard seed oil on the basic functional properties, flammability, and biodegradation of rigid polyurethane–polyisocyanurate foams, which were obtained as a result of partial replacement of petrochemical polyol by vegetable-based polyol. Furthermore, the effect of bio-polyol on the properties of materials was examined after the elimination of a commercial flame retardant containing chloroorganic phosphates. These compounds are considered harmful to the environment. Therefore, new solutions are sought to reduce or eliminate them from use in the polyurethane industry. The obtained results were also compared with the parameters of commercial polyurethane–polyisocyanurate foam used as industrial thermal insulation materials.

## 2. Materials and Methods

### 2.1. Materials

**Figure 1:**
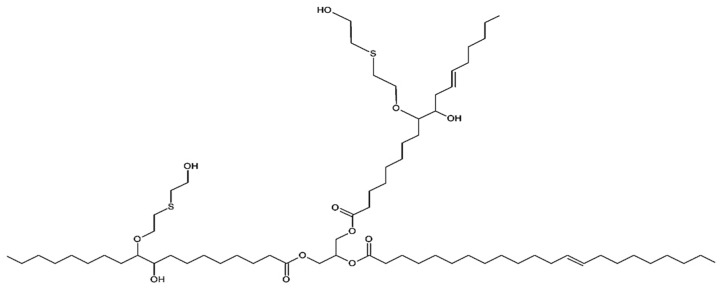
Schematic chemical structure of PG1 bio-polyol.

White mustard seed oil-based bio-polyol and 2,2′-thiodiethanol (3-thiapentane-1,5-diol) with hydroxyl number HN = 291.83 mg KOH/g (produced in accordance with Pat. PL-233221 in Department of Chemistry and Technology of Polyurethanes, Kazimierz Wielki University, Bydgoszcz, Poland [23,24]) was used in the mixture with petrochemical polyol Rokopol RF-551 (sorbitol oxyalkylation product; hydroxyl number HN = 420 mg KOH/g; produced by ZCh PCC Rokita SA, Brzeg Dolny, Poland) for the synthesis of rigid polyurethane–polyisocyanurate (RPU/PIR) foams. The used bio-polyol was obtained from cold-pressed white mustard seed oil in two-step synthesis (epoxidation and ring-opening reactions) and was marked with the abbreviation PG1. The detailed description of the synthesis (step by step), based on the cited patent, was described in [24,25,26]. Detailed properties of this raw material are in [24]. The schematic chemical formula of the bio-polyol (based on nuclear magnetic resonance (NMR) spectroscopy analysis, Figures S1–S6) is shown in Figure 1.

Purocyn B, a technical polyisocyanate (supplied by Purinova Ltd., Bydgoszcz, Poland), was used as the isocyanate raw material. The main component was 4,4’-diphenylmethane diisocyanate. The content of free NCO groups was equal to 31%.

The catalytic system for synthesis of RPU/PIR foams was made from anhydrous potassium acetate (produced by Chempur, Piekary Śląskie, Poland) used in a 33% solution of diethylene glycol (produced by Chempur, Piekary Śląskie, Poland). DABCO, a 1,4-diazabicyclo[2.2.2]octane (produced by Alfa Aesar, Haverhill, MA, USA), was also used in a 33% solution of diethylene glycol. Tegostab 8460, a polysiloxanepolyoxyalkylene surfactant (produced by Evonik, Essen, Germany), was used as a foam structure stabilizer. Solkane HFC 365/227, a mixture of 1,1,1,3,3-pentafluorobutane and 1,1,1,2,3,3,3-heptafluoropropane in a mass ratio of 87:13 (produced by Solvay, Brussels, Belgium), was used as a blowing agent. Antiblaze TCMP, a tri(chloro-2-methylethyl) phosphate (produced by Albemarle, Charlotte, NC, USA), was used as a flame retardant.

### 2.2. Preparation of RPU/PIR Foams

The formulation of RPU/PIR foams with the addition of white mustard seed oil-based bio-polyol required experimental research to determine the optimal composition of additives (catalysts, surfactant, flame retardant, and blowing agent). The value of hydroxyl number was a base for the calculation of the polyol raw materials amount. The aforementioned value was used to calculate the quantities of polyols [25,26]. During the synthesis of foams, the petrochemical polyol was partially replaced by a mustard seed oil-based bio-polyol in an amount from 0 to 0.4 of chemical equivalent (*Eq*) of hydroxyl groups (every 0.1 *Eq*). The amount of isocyanate raw material was calculated regarding the chemical equivalent (*Eq*) ratio of the NCO to hydroxyl (OH) groups in the reaction mixture, which was found to be 3:1 for RPU/PIR foams. The trimerization of the three NCO groups to the isocyanurate ring is necessary, as well as for the reaction between the NCO and OH groups to produce a urethane bond. The sum of the chemical equivalents of petrochemical polyol and mustard seed oil-based bio-polyol was always 1. The amount of additives was determined, respectively, by the catalyst for the reaction between NCO and OH groups (1 wt.%, the trimerization catalyst, 2.5 wt.%, the physical blowing agent, 12 wt.%, flame retardant, 17 wt.% and surfactant, and 1.7 wt.% in relation to the mass sum of polyols and polyisocyanate). Two series of foams modified with bio-based polyol were obtained—with commercial flame retardant (ZA) and without it (BA). The formulation of polyurethane–polyisocyanurate foams is shown in Table 1.

The foams were obtained on a laboratory scale using the one-step method from the two-component (A and B) system. Component A was obtained from mixing appropriate amounts of polyols, catalysts, surfactant, blowing agent, and, optionally, flame retardant. Component B was a technical polyisocyanate, Purocyn B. The components A and B were mixed for 10 s with a mechanical stirrer (1800 rpm) in a required mass ratio (Table 1). Next, the mixture was poured into a rectangular open mold with internal dimensions of 25 cm × 25 cm × 30 cm, where the free rising of foam took place. The progress of the foaming process was analyzed by an electronic stopwatch to determine the characteristic foaming times. Foams were thermostated for 4 h at 120 °C in a laboratory dryer with forced circulation after removing from the mold.

### 2.3. Characterization of RPU/PIR Foams

The functional properties of obtained RPU/PIR foams were determined in accordance with the standards for rigid polyurethane foams.

#### 2.3.1. Processing Times

Characteristic foaming time was analyzed during the process using an electronic stopwatch in accordance with ASTM D7487 13e^1^. Cream, free-rise, string gel, and tack-free times were measured during synthesis of the RPU/PIR foams [27].

#### 2.3.2. Physico-Mechanical Properties 

The apparent density of foams (the ratio of foam weight to its geometrical volume) was determined for cube-shaped samples with a side length of 50 mm in accordance with ISO 845:2006.

The compressive strength was determined using the universal testing machine Instron 5544 (Instron, Norwood, MA, USA) in accordance with ISO 844:2014. The maximum force inducing a 10% relative strain was determined (decreasing of the foam height in relation to the initial height, according to the direction of foam rise).

The brittleness of the foams was determined in accordance with ASTM C-421-08 as a percentage mass loss of 12 cubic foam samples with a side length of 25 mm. Tests were conducted in a standard cuboidal box made of oak wood with dimensions of 190 mm × 197 mm × 197 mm, rotating around the axis at a speed of 60 rpm. The filling of the box during the measurement were 24 normalized oak cubes with dimensions of 20 mm × 20 mm × 20 mm. The brittleness (B) of obtained foams was calculated from Equation (1):

(1) $$B=\frac{m_{1}-m_{2}}{m_{1}}\cdot 100\%$$

where *m*_1_—mass of the sample before test (g) and *m*_2_—mass of the sample after test (g).

Absorbability (*A*) and water absorption (*WA*) were determined in accordance with ISO 2896:2001, which was measured after immersion in distilled water for 24 h. Values of these parameters were calculated from Equations (2) and (3):

(2) $$A=\frac{m_{A}-m_{D}}{m_{D}}\cdot 100\%$$

where *m_A_*—mass of the sample after immersion in distilled water (g) and *m_D_*—mass of the dry sample (g).

(3) $$WA=\frac{m_{WA}-m_{D}}{m_{D}}\cdot 100\%$$

where *m_WA_*—mass of the sample after surface drying (g).

#### 2.3.3. Aging Resistance Properties

Aging resistance of the foams was carried out in thermostating process of cubic samples with a side length of 50 mm for 48 h at a temperature of 120 °C. The result of this test included a change of linear dimensions (Δ*l*), change of geometrical volume (Δ*V*), and mass loss (Δ*m*). The values of these parameters were calculated in accordance with ISO 1923:1981 and PN-EN ISO 4590:2016-11. The formulas for the calculations of Δ*l*, Δ*V*, Δ*m* are shown in Equations (4)–(6).

(4) $$\Delta l=\frac{l-l_{0}}{l_{0}}\cdot 100\%$$

where *l*_0_—length of the sample before thermostating (according to the direction of foam rise) (mm) and *l*—length of the sample after thermostating (according to the direction of foam rise) (mm).

(5) $$\Delta V=\frac{V-V_{0}}{V_{0}}\cdot 100\%$$

where *V*_0_—geometrical volume of the sample before thermostating (mm^3^) and *V*—geometrical volume of the sample after thermostating (mm^3^).

(6) $$\Delta m=\frac{m_{0}-m}{m_{0}}\cdot 100\%$$

where *m*_0_—mass of the sample before thermostating (g) and *m*—mass of the sample after thermostating (g).

#### 2.3.4. Cell Structure

The foam structure was analyzed by scanning electron microscope (SEM) HITACHI SU8010 (Hitachi High-Technologies Co., Tokyo, Japan). The studies were performed at the accelerating voltage of 30 kV, with the working distance of 10 mm and magnification of 150x. The statistical analysis of cell sizes, wall thickness, and content of cell per area unit was carried out on the basis of obtained micrographs using ImageJ software (LOCI, Madison, WI, USA).

#### 2.3.5. Thermal Insulation Properties

Thermal conductivity of the foams was determined based on the determination of the thermal conductivity coefficient λ in accordance with ISO 8301. Tests were carried out with the FOX 200 apparatus (TA Instruments, New Castle, DE, USA), in the measurement range of λ equal to 20–100 mW/(m·K). Measurements were performed in the series at intervals of 0.5 s and at an average measuring temperature of 10 °C (temperature of hot plate—20 °C, temperature of cold plate—0 °C).

The content of closed cells was determined in accordance with PN-EN ISO 4590:2016-11 using the helium pycnometer AccuPyc 1340 with the FoamPyc (Micrometrics, Norcross, GA, USA). This software calculated the content of closed cells based on the measurement of pressure changes in the test chamber.

#### 2.3.6. Flammability Tests

The flammability of RPU/PIR foams was determined using four flammability tests: Bütler’s combustion test (vertical test) in accordance with ASTM D3014-04, the horizontal combustion test in accordance with PN-EN ISO 3582:2002/A1:2008, limiting oxygen index in accordance with ISO 4589, and microcalorimeter pyrolysis combustion flow calorimeter (PCFC) in accordance with ASTM D7309-2007.

Bütler’s combustion test consisted of burning a foam sample with the dimensions of 150 mm × 20 mm × 20 mm in a vertical column (chimney) with dimensions of 300 mm × 57 mm × 54 mm. Combustion residue (CR) was calculated from Equation (7):

(7) $$CR=\frac{m_{b}}{m_{a}}\cdot 100\%$$

where *m_a_*—mass of the sample before the burning test (g) and *m_b_*—mass of the sample after the burning test (g). 

The horizontal burning test consisted of determining the susceptibility of foam to flame. The result of this test was an evaluation of material flammability (nonflammable, flammable, or self-extinguishing). The average burning rate in flame (BR) of the tested materials was also determined based on this test. This parameter was defined by an equation (8):

(8) $$BR=\frac{s}{t}$$

where *s*—the distance, which was passed by flame during measurement (mm), and *t*—time from the beginning of the measurement to the self-extinguishing of the flame (s). 

The limiting oxygen index (LOI) was measured using the Concept Equipment apparatus (Rustington, UK). The percentage limited concentration of oxygen was determined in the mixture consisting of oxygen and nitrogen, which was sufficient to sustain the burning of the sample. LOI was calculated according to the Equation (9).

(9) $$LOI=\frac{\left[O_{2}\right]}{\left[O_{2}\right]+\left[N_{2}\right]}\cdot 100\%$$

where *[O_2_]*—volumetric flow of oxygen at the limit concentration (m^3^/h) and *[N_2_]*— olumetric flow of nitrogen at the limit concentration (m^3^/h).

The fourth flammability test was the pyrolysis of RPU/PIR foams using a PCFC microcalorimeter (Fire Testing Technology, East Grinstead, UK). A microcalorimeter is a device designed to determine the rate of heat release (HRR) by materials. The test method is based on measuring heat and oxygen depletion during the thermal decomposition of a small sample of material. The decomposition is carried out in an inert atmosphere in the temperature range of 150–750 °C with a heating rate of 1 °C /s. Then, the gases formed as a result of pyrolysis are oxidized in a high-temperature furnace at 900 °C for complete oxidation. Parameters, such as time to ignition (TTI), time to flameout (TTF), heat release rate (HRR), and total heat released (THR), were determined by this device.

#### 2.3.7. Susceptibility to Biodegradation

The study of biodegradation process RPU/PIR foams modified by mustard seed oil-based bio-polyols was carried out in accordance with ISO 17556:2012 using the OxiTop Control S6 apparatus (WTW-Xylem, Rye Brook, NY, USA), which used a respirometric method to measure the oxygen demand necessary for aerobic biodegradation of polymeric materials in soil. The measurement of consumed oxygen was presented using the value of biochemical oxygen demand (BOD), which is the number of milligrams of captured oxygen per mass unit of tested polyurethane material.

The OxiTop Control S6 apparatus consisted of six glass bottles with a capacity of 510 mL equipped with rubber quivers and measuring heads, which were used to measure BOD. They allowed us to measure the pressure in the range of 500 hPa to 1350 hPa with an accuracy of 1% at a temperature of 5 °C to 50 °C. The apparatus also included the OC 110 controller (WTW-Xylem, Rye Brook, NY, USA), It was used for communication between the measuring heads, user, and the Achat OC computer software (WTW-Xylem, Rye Brook, NY, USA), which was used to interpret the obtained measurement results.

Sifted and dried garden soil with a high humus content and physicochemical parameters, such as humidity of 5% (according to ISO 11274), pH of 6 (according to ISO 10390), and grain diameter below 2 mm (collected in Szczepanowo, Kuyavian-Pomeranian Voivodeship, Poland), was used as a biodegradation environment. The measurement was carried out in a system consisting of 200 mg of tested foam, 200 g of soil, and 100 g of distilled water. The hermetic-closed system was placed in a laboratory incubator at 20 °C ± 0.2 °C and thermostated at this temperature for 28 days. All tested samples were comparable in size, because the absolute degree of polymer degradation depends on the form and shape of the material [28].

The BOD for a single OxiTop Control S6 bottle was determined from Equation (10) taking into account the BOD of the tested system reduced by the BOD of the soil and concentration of the tested material in the soil.

(10) $$BOD_{S}=\frac{BOD_{x}-BOD_{g}}{c}$$

where S—number of measurement days, BOD_S_—biochemical oxygen demand of the analyzed material within S days (mg/L), BOD_x_—biochemical oxygen demand of the measuring system (bottle with sample and soil) (mg/L), BOD_g_—biochemical oxygen demand of soil without a sample (mg/L), and c—sample concentration in the tested system (mg/L). 

The degree of biodegradation of the polymeric material was determined based on Equation (11):

(11) $$D_{t}=\frac{BOD_{S}}{TOD}\cdot 100\%$$

where D_t_—degree of polymer biodegradation (%) and TOD—theoretical oxygen demand (mg/L). The theoretical oxygen demand for each system was calculated from Equation (12):

(12) $$TOD=\frac{16\left[2c+0.5\left(h-cl-3n\right)+3s+2.5p+2f+0.5k-o\right]}{m_{r}}$$

where c, h, p, s, n, cl, f, k, o—mass shares of elements in the macromolecule of biodegradable material (-) and m_r_—sample mass of biodegradable material (g).

## 3. Results and Discussion 

### 3.1. Foaming Process and Reactivity of Bio-Polyol

Processing times were measured during the preparation of RPU/PIR foams to determine the effect of bio-polyol on foaming parameters, i.e., cream time—from the start of mixing components A and B until fine bubbles appeared; free-rise time—from the start of mixing components A and B until the foam stops expanding; string gel time—from the start of mixing components A and B until long strings of tacky material can be pulled away from foam surface when the surface is touched by a tongue depressor; tack-free time—from the start of mixing components A and B until the foam surface can be touched by a tongue depressor without sticking. All foams retained the assumed percentage of additive raw materials (Table 1) for easier interpretation of the obtained results. The results of measurements are shown in Table 2. In both cases, the foams featured a tendency to elongate. A clear increase in processing times was noted after the use of bio-polyol in presence of commercial flame retardant (ZA series). This effect implied that the mustard oil-based polyol had lower reactivity than the petrochemical polyol. This is consistent with literature reports on other bio-polyols, e.g., based on rapeseed, soybean, sunflower, or palm oil [29,30,31].

In the case of BA series foams, changes were not observed in the cream, string gel, and tack-free times. However, the free-rise times were slightly longer, meaning that the lower reactivity of the bio-polyol did not significantly affect the rate of the foaming process, despite materials obtained without commercial flame retardant having higher viscosity than the polyol premixes (component A). The elimination of flame retardant from the polyurethane formulations did not extend the processing times, which remained at the level of the reference foam.

### 3.2. Physico-Mechanical Properties

**Figure 2:**
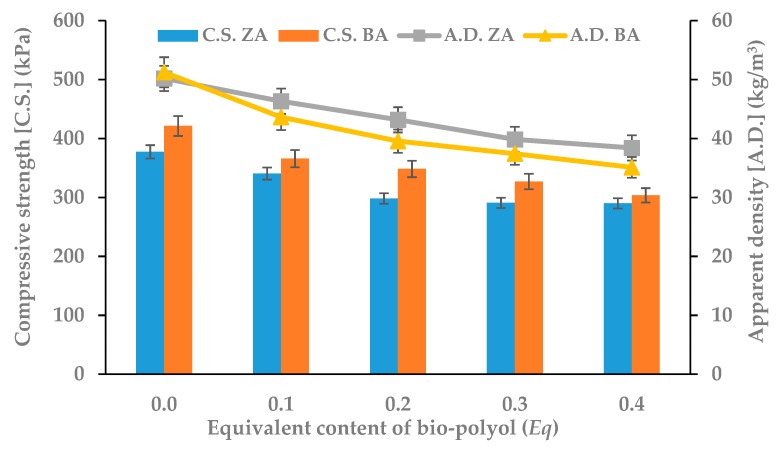
Dependence between bio-polyol content, compressive strength (C.S.) and apparent density (A.D.).

An important parameter determining the use RPU/PIR foams as a thermal insulation material is their apparent density. It indirectly influences other physical and mechanical properties, e.g., compressive strength or brittleness [32,33]. Increasing the content of mustard seed oil-based bio-polyol decreased the apparent density of ZA series foams by 20% for 0.4 *Eq* and BA series foams by 30% in comparison with appropriate reference foams. The decrease in the degree of polymer macromolecule packing was caused the addition of a component, which contained long, linear chains, to the polyurethane (Figure 1). It proved that fatty acid residues were flexible segments with low cross-linking potential. This phenomenon also significantly affected the compressive strength of rigid foams [34,35,36]. This parameter decreased with the increasing content of bio-polyol by 25% for ZA series and 30% for BA series. The main reason for the decrease was the increasing content of flexible segments in the polyurethane matrix. The dependence between apparent density, compressive strength, and bio-polyol content is shown in Figure 2. Despite the high decrease in apparent density and compressive strength, the values of these parameters remained at a satisfactory level compared to the material offered commercially (compressive strength higher than 250 kPa) [37]. 

**Figure 3:**
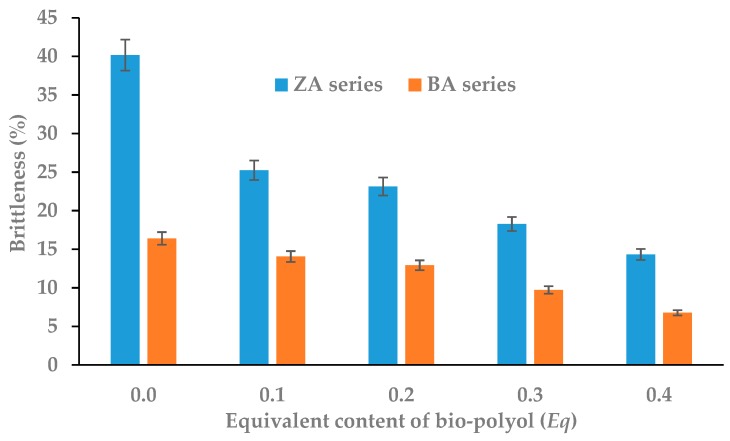
Dependence between bio-polyol content and brittleness of RPU/PIR foams.

Another consequence of the decrease in the degree of polymer macromolecule packing was a slight decrease of stiffness and increase of flexibility, which led to a significant reduction in the brittleness of tge obtained rigid RPU/PIR foams. When a white mustard seed oil-based bio-polyol was used, the decrease of this parameter was more than 60% for the P1.4 ZA foam and 50% for the P1.4 BA foam (Figure 3). The reduction of brittleness for the rigid polyurethane–polyisocyanurate foams meant an increase in resistance to mechanical factors and an increase in adhesion to various surfaces [38,39].

**Figure 4:**
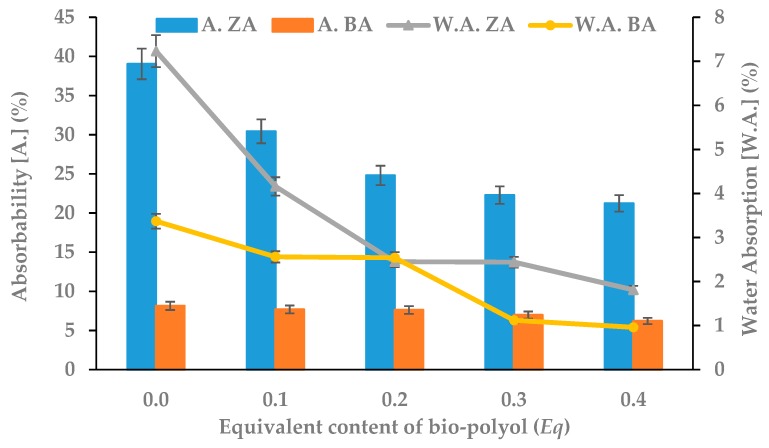
Dependence between bio-polyol content, absorbability (A.) and water absorption (W.A.).

An important parameter of porous thermal insulation materials is their absorbability and water absorption. The first parameter is the content of water in the material immediately after being taken out of the immersion (inside and on the foam surface). The second is a percentage value of water remaining inside the foam [40,41,42]. In both cases, a visible reduction in these parameters was noted (Figure 4). The decrease of absorbability and water absorption in foams based on bio-polyol was mainly caused by the addition of hydrophobic groups derived from vegetable oil into the polyurethane matrix. Moreover, the obtained results showed that the synthesized RPU/PIR foams met the requirements for commercially offered materials. The values of absorbability and water absorption allow them to be used as thermal insulating materials in high-humidity environments [43].

Obtained results were in the range of values assumed for this type of polyurethane materials. Table 3 presents a comparison of foams with the highest content of white mustard oil-based bio-polyol with commercial RPU/PIR foam.

### 3.3. Aging Resistance Properties of RPU/PIR Foams

Aging resistance tests, i.e., stability of linear dimensions (Δl) and change of geometrical volume (ΔV), showed that foams modified with bio-polyol up to 0.4 *Eq* had these parameters at the same level as the reference foams, respectively, of 0.90% and 2.60% for the ZA series and 0.97% and 2.70% for the BA series. The increased content of long elastic segments allowed for an easier release of the blowing agent from foams (mixture of 1,1,1,3,3-pentafluorobutane and 1,1,1,2,3,3,3-heptafluoropropane - Solkane HFC 365/227) [44]. However, no changes were observed in the structure of the material indicating the increased diffusion of gases from the interior of the RPU/PIR foams.

Studies of mass loss after aging showed that the addition of the mustard oil-based bio-polyol caused the reduction of this parameter from 5.91% (ZA) and 2.88% (BA) for reference foams to 2.93% (ZA) and 1.25% (BA) for foams with 0.4 chemical equivalent of this raw material, meaning that the bio-polyol had a higher aging resistance than the petrochemical polyol because the oleochemical raw materials have better stability at higher temperature [45]. 

### 3.4. Foam Structure

**Figure 5:**
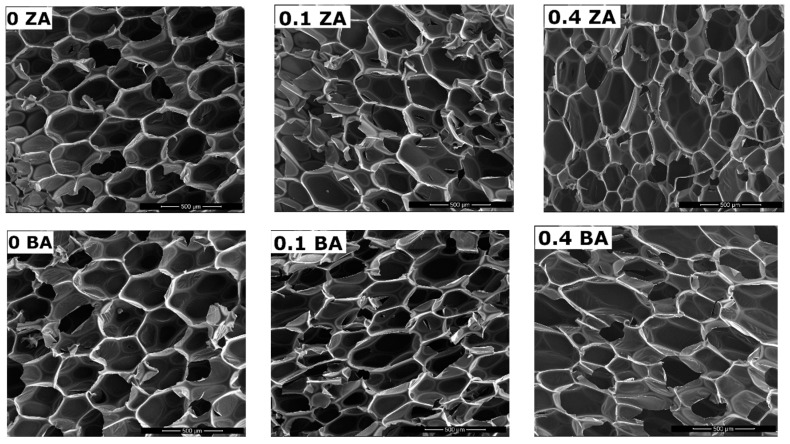
Micrographs of cellular structure of foam with 0, 0.1, and 0.4 *Eq* of bio-polyol in 150x (ZA series on the top, BA series bottom).

In case of using plant-based polyols for the production of rigid polyurethane and polyurethane/polyisocyanurate foams, their influence on the shape and size of the cells of foam was noted. The use of mixtures of petrochemical polyol and soybean-based bio-polyol resulted in obtaining polyurethane materials with larger cell diameters than in materials without the participation of this bio-polyol [46]. On the other hand, using a bio-polyol based on castor oil and raw glycerol in a mixture with petrochemical polyol caused a beneficial influence on the cellular structure of RPU/PIR foams. There was a decrease in average cell diameter from 372 μm to 275 μm and an increase in the content of closed cells [47]. The cellular structure was examined for: reference foams, foams without mustard seed oil-based bio-polyol (P1.0 series ZA and BA), foams containing 0.1 chemical equivalent of the bio-polyol (P1.1), and foams with the highest content of the bio-polyol (P1.4). Based on micrographs presented on Figure 5, it was found that the increase in bio-polyol content caused an increase in cell diameters and a slight increase in wall thickness. The increase of bio-polyol content also caused a decrease in the symmetry of the cell structure. The average cell size, average wall thickness, and content of cells per volume unit were determined based on the SEM micrographs. The obtained results are showed in Table 4. 

The main factor that caused changes in foam structure was the addition of long linear molecules (fatty acid glycerides) of polyol. High flexibility of polyol segments and lower crosslinking (lower hydroxyl number than in commercial polyol) allowed greater migration of the blowing agent. As a result, the obtained foams had larger cell diameters and thicker walls. This was indirectly influenced by other RPU/PIR properties such as apparent density, compressive strength, and brittleness [48,49,50].

### 3.5. Thermal Insulation Properties

**Figure 6:**
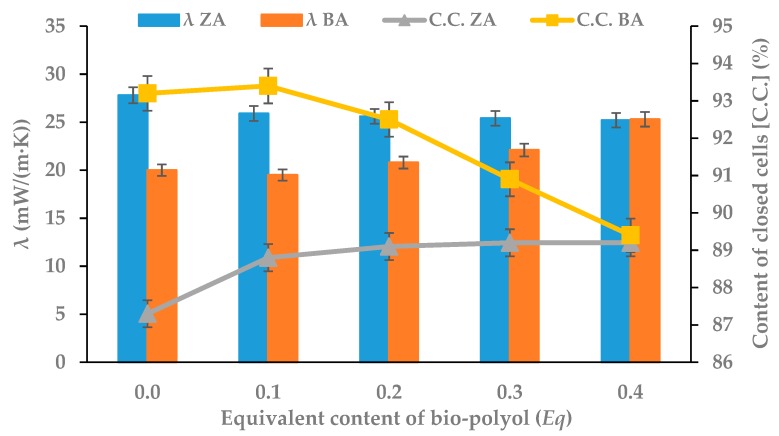
Dependence between bio-polyol content, thermal conductivity coefficient λ and content of closed cells (C.C.).

The most important parameter of the polyurethane materials dedicated for used as thermal insulation is λ, the thermal conductivity coefficient. If its value is lower, then the material is a better heat insulator [51]. With regard to foams obtained with the increasing content of bio-polyol, it was noted that the presence of a flame retardant had a significant effect on their thermal conductivity coefficient and insignificant effect on content of closed cells (Figure 6).

The BA reference foam showed a 30% lower coefficient λ than the ZA reference foam. This was due to the more compact structure of the polyurethane matrix. Among the rigid PUR macromolecules were chemically unbound molecules of tri(2-chloro-1-methylethyl) phosphate, which disturbed the diffusion of heat during the measurement. The addition of molecules containing long partially bound aliphatic chains (fatty acid residues in bio-polyol) allowed for better diffusion of heat in the material. Hence, the noticeable increase of the λ coefficient occurred in the BA foam series. The case of the ZA series was different. Alongside unbound molecules of flame retardant, there were also aliphatic chains which were partially connected with polyurethane matrix. Their mutual presence interfered with internal vibrations of molecules in polyurethane matrix, which caused a worse diffusion of heat through the material. Hence, a decrease in the value of this coefficient for ZA series was observed. In both cases, foams with the highest bio-polyol content had a lower λ value than commercially heat insulation material, which was 0.028 W/(m·K) [37].

The content of closed cells in foams is as important as the thermal conductivity coefficient. An increase in the value of this parameter causes a decrease in the λ value. It is known that the share of thermal conductivity of the gas contained in foam cells is about 60–80% of the total value of the thermal conductivity coefficient of polyurethane foam. The share of thermal conductivity depends primarily on the presence and type of blowing agent in the cells and the apparent density of the foam. The tendency to open cells and release blowing agent causes an increasing the thermal conductivity coefficient [52]. Based on the results presented in Figure 6, it can be seen that the content of closed cells increased from 87.3% (0 *Eq*) to 89.2% (0.4 *Eq*) for foams of the ZA series and decreased from 93.2% (0 *Eq*) to 89.4% (0.4 *Eq*) for BA series foams. Changes in the value of this parameter are small enough to fall within the permissible industrial standards. Therefore, it can be concluded that the use of white mustard oil-based bio-polyol and a commercial flame retardant does not have a significant influence on the change of closed cell content in RPU/PIR foams.

### 3.6. Flammability of RPU/PIR Foams 

The ease of ignition and release of large amounts of smoke during a fire, as well as the release of toxic gases, are significant difficulties associated with the use of polyurethanes. Increasing the flame retardancy of these materials is necessary to avoid safety issues and to comply with increasingly stringent legal requirements for flammability of plastics. Safety issues necessitate the use of thermally stable and nonflammable materials, which release little heat and toxic gases. Polyurethanes undergo thermal decomposition at high temperatures. High temperature, smoke, and volatile substances are produced at the beginning of the polyurethane combustion process. The last compounds consist of a mixture of flammable and nonflammable volatile substances such as monomers, hydrocarbons, carbon monoxide, and carbon dioxide. The actual quantity of produced volatile substances depends on the chemical structure and additive agents used in the polyurethane formulation. Flammable volatile substances react with oxygen to form of highly reactive hydrogen and hydroxide radicals, which accelerate further thermal decomposition and burning of polyurethane. Polyurethanes may degrade completely during thermal decomposition or may form charred or inorganic residues, depending primarily on the ingredients used in the formulation. The use of flame retardants can increase the time to ignition of polyurethane, reduce or eliminate the flame combustion phase, and reduce the heat release rate and flame propagation on the material surface [53].

The effect of flame-retardant compounds is usually considered from the point of view of reactions, which occur in the flame, and the formation of a boundary layer with different insulating properties. It is optimal to choose flame-retardant compounds that affect both of these phases (gas phase and condensed phase) [54]. A chemical modification based on the synergy effect of sulfur heteroatom with chlorine and phosphorus compounds was used as a part of this research. This modification consisted of the addition of these elements into the polyurethane foam matrix during their preparation.

**Figure 7:**
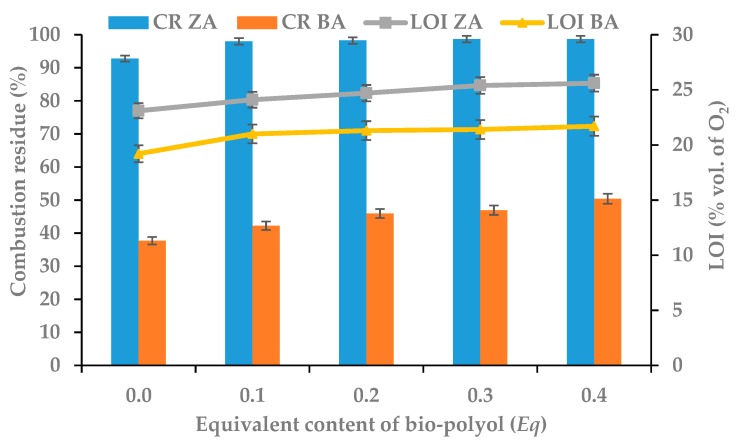
Dependence between bio-polyol content, combustion residue (CR) and limiting oxygen index (LOI).

The flammability tests of the obtained RPU/PIR foams were carried out by four methods: Determining the residue after combustion (CR) in the vertical test, determining the limiting oxygen index (LOI), determining the material classification according to PN-EN ISO 3582:2002/A1:2008, and determining a fire parameters using PCFC calorimeter. These methods made it possible to compare the results of flammability of materials. This comparison was also possible after their modification using bio-polyol containing sulfur heteroatoms or the addition of a commercial flame retardant, Antiblaze TCMP. Changes in the values of combustion residue and limiting oxygen index of RPU/PIR foams with different content of white mustard oil-based bio-polyol oil and with or without flame retardant are presented in Figure 7.

Rigid polyurethane foams that do not contain flame retardants have a LOI value of up to 20%. Therefore, they are flammable materials. The P1.0 BA reference foam, a foam obtained using only commercial polyol which did not contain a flame-retardant compound, had a LOI value of 19.2%. Foams from P1.1 BA to P1.4 BA were obtained using plant-based polyol, which contained a sulfur heteroatom. The increasing content of this bio-polyol in the polyurethane formulation (increasing the percentage amount of sulfur) caused an increase in the LOI value to 21.7% for the foam with the highest content of it. However, the use of the flame-retardant compound in the formulation (ZA series of foams) resulted in a significant reduction of the flammability of obtained materials. This was confirmed by the results of the limiting oxygen index, which showed values increasing from 23.1% for the reference foam to 25.6% for the foam with the highest content of mustard-oil based bio-polyol containing sulfur heteroatoms. The obtained RPU/PIR foams modified with vegetable-based polyol and a flame-retardant compound showed significantly higher LOI values in the range of 21–26%. These results classified them as slow-burning materials. It should be noted that the conducted research concerned the preparation of modified polyurethane/polyisocyanurate foams, which contained isocyanurate rings obtained by increasing the isocyanate index [55,56]. The presence of isocyanurate rings promoted the formation of a charred layer on the surface of the burning foam. This charred layer was formed from volatile flammable gases and acted as a protective barrier by sealing the polymer against oxygen and preventing the release of volatile products of polymer pyrolysis. Sulfur added into the polyurethane structure was transformed into sulfenic and thiosulfoxylic acids in the process oxidation of sulfide bonds. Then, it was gradually transformed into SO_2_ and SO_3_. These acids were the catalysts for ionic peroxide decomposition, while sulfur oxides inhibited chain reactions of oxidation. Thus, these compounds exerted a flame-retardant effect in the gas phase [57,58], while the chloro-phosphorus organic compound had a flame-retardant effect in the condensed phase. The effectiveness of its operation consisted in creating a protective layer on the polymer surface hindering the spread of fire and heat transfer to further layers of the polymer. The flame-retardant compound was decomposed to simpler compounds that were involved in oxidation reaction by free radicals under the influence of heating. Then, there was a decrease in the concentration of active free radicals, which slowed down the combustion process [59,60,61,62]. The reason for the reduction of flammability of obtained RPU/PIR foams was the synergism of sulfur contained in the bio-polyol with chlorine and phosphorus contained in flame retardant (Antiblaze TCMP). Similar synergism was observed by Bhoyate et al. [63,64]. They confirmed the active flame-retardant effect in the combination of phosphorus containing compounds with natural raw materials such as soybean oil, castor oil, and limonene. 

The limiting oxygen index results confirmed the values of combustion residue for the obtained RPU/PIR foams. Figure 7 shows a clear difference of this parameter for RPU/PIR foams containing bio-polyol and for reference foams. Materials based on bio-polyol were characterized by much higher combustion residues than unmodified foams. Even a small amount of this bio-polyol in the foam formulation significantly increased their CR (an increase of 6% for foam of ZA series and an increase of 13% foam of BA series). It is worth noting that the addition of a commercial flame-retardant compound contributed to a significant increase in the combustion residue for foams of the ZA series from 92.70% (P1.0) to 98.67% (P1.4) and for foams of the BA series from 37.72% (P1.0) to 50.39% (P1.4). Another reason for this phenomenon was the synergism of sulfur, chlorine, and phosphorus, which were components of flame retardant and bio-polyol incorporated into the polyurethane matrix.

**Figure 8:**
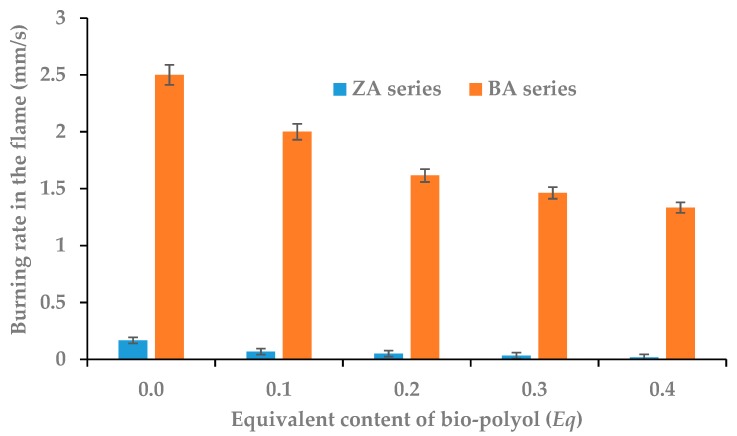
Dependence between bio-polyol content and burning rate in flame (BR).

**Figure 9:**
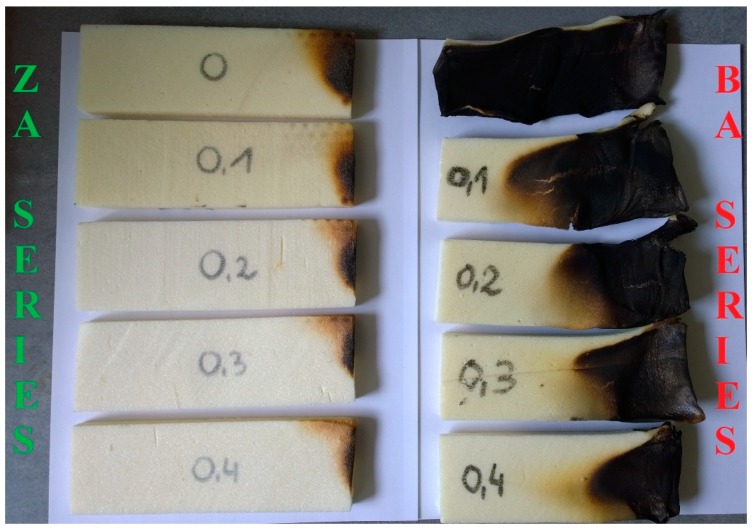
Appearance of ZA and BA series of foams after horizontal burning test.

The horizontal flammability test showed that all foams of the ZA series were self-extinguishing. In the case of foams of the BA series, the increase of bio-polyol content resulted in a change in material classification. The reference foam (P1.0 BA) was classified as a flammable material. In contrast, the foam with the highest content of bio-polyol was already self-extinguishing. The burning rate of material in flame (BR) was also determined based on this test. The results are shown in Figure 8, while appearance of foams after burning test is shown in Figure 9.

The burning rate in the flame of the ZA series foams was low. However, the increase in bio-polyol content caused a 10-fold decrease of this parameter from 0.167 mm/s to 0.017 mm/s, meaning that the P1.4 ZA foam practically did not burn. The burning rate in the flame of the BA series foams was much higher. This was obviously due to the lack of a commercial flame retardant. For the reference foam, the burning rate in the flame was 2.500 mm/s. The value of this parameter was almost half for foam with the highest content of mustard oil-based bio-polyol and 2,2’-thiodiethanol (P1.4 ZA), which was 1.330 mm/s. The obtained results confirmed the previously described effect of sulfur and flame retardant on flammability of RPU/PIR foams. 

Significant information about flammability of RPU/PIR foams was provided by the PCFC microcalorimeter. However, it should be noted that the PCFC method used milligram quantities of polymer samples, and the analysis was carried out under specific conditions which do not reflect real fire conditions. Nevertheless, it is a commonly used method for the flammability assessment of various materials modified with flame retardants. These results are presented in Table 5.

**Figure 10:**
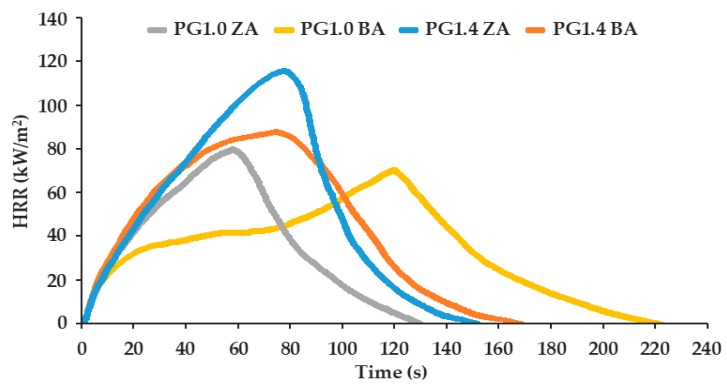
Heat release rate (HRR) as a function of time for RPU/PIR foams.

One of the most important flame-retardancy parameters is the time to ignition (TTI) of the test sample. If this time is long, then the material will heat up longer and ignite longer. Then, the probability of fire is lower. Based on the obtained results, the P1.0 BA foam showed a very short ignition time (TTI = 6 s), which is typical for materials with a porous structure (characterized by high flammability). The addition of flame-retardant compound in the P1.0 ZA foam formulation caused a slight increase of this time to 10 s, while the RPU/PIR foams obtained on the basis of plant-based bio-polyol began to burn after 12 s and after 21 s for the P1.4 BA and the P1.4 ZA foams, respectively. It was easy to see that the addition of commercial flame retardant to the formulation containing bio-polyol increased the time to ignition more than two-fold. The time to flameout (TTF) was the longest for the P1.0 BA foam (175 s), while the heat was released slowly. On the other hand, the use of chloro-phosphorus flame retardant in a mixture with bio-polyol significantly reduced the time to flameout to 69.4 s for the P1.4 ZA foam. It also had an influence on the course of curves of heat release rate (HRR) of foams as a function of time (Figure 10).

The foam obtained from a petrochemical polyol, which did not contain a flame retardant (P1.0 BA), was characterized by a lower HRR than other foams, which were obtained from bio-polyol and with or without flame retardant. It should be noted that these foams reached this rate in shorter time (after about 50 s), while the P1.0 BA foam ignited quickly but burned slowly. It also released heat slowly, and reached its maximum burning rate after a longer time (120 s). The P1.4 ZA foam reached the highest value of the HRR, which was 114.8 kW/m^2^. The P1.0 BA foam had an HRR value of about 60 kW/m^2^. The addition of a flame-retardant compound to the obtained foams also affected the total heat released (Table 5). The reduction of THR was visible after addition of the bio-polyol and the chloro-phosphorus flame retardant into the polyurethane composition. These values were from 23.1 MJ/m^2^ for the P1.0 BA foam to 9.7 MJ/m^2^ for the P1.4 ZA foam. The flammability reduction, which can be observed for foams modified by mustard-based polyol and Antiblaze TCMP, was due to the introduction of sulfur heteroatom, as well as chlorine and phosphorus atoms, into the polyurethane. The chain reaction of free radicals formed in the gaseous phase was interrupted during the combustion process. This resulted in the dilution of gaseous products and the extinguishment of the flame. In addition, the flame-retardant effect was increased with increasing the sulfur content due to aforementioned synergistic effect. The combined effect of bio-polyol and flame-retardant compound in condensed and gas phases resulted in increased flame retardancy of the obtained RPU/PIR foams.

### 3.7. Susceptibility to Biodegradation 

A very important problem of the modern world is the faster and faster increase in the consumption of polymeric materials, and thus the increasing emission of waste. This problem is especially visible in the case of RPU/PIR foams. These materials are much more resistant to degradation due to the presence of isocyanurate rings in their structure. The presence of this group also causes their waste to be very persistent in the environment (the natural decomposition lasts hundreds or thousands of years). RPU/PIR foams waste can be chemically processed, e.g., in a glycolysis process. However, full-valued products cannot be obtained, such as before their synthesis. The obtained glycolysates can only constitute an admixture to formulations of RPU/PIR foams because they frequently affect the deterioration of all performance properties of materials [5,65,66].

Another reason for the high resistance of RPU/PIR foams to environmental factors is the presence of petrochemical raw materials (mainly carbon of petrochemical origin) in their composition. They are much less degradable in the environment than raw materials of plant origin [67]. Therefore, it was examined how the modification of the smallest (0.1 *Eq*) and the highest (0.4 *Eq*) content of white mustard seed oil-based bio-polyol would affect the biodegradation process of RPU/PIR foams in soil. For this purpose, the share of individual elements in RPU/PIR foams subjected to the biodegradation process was determined based on literature and experimental data (elemental analysis of raw materials). The obtained results are shown in Table 6.

The results presented in Table 6 showed that elements such as carbon, hydrogen, oxygen, nitrogen, and sulfur underwent fundamental changes in RPU/PIR foams after modification by bio-polyol. The amounts of other elements constituting the additives (e.g., potassium from the trimerization catalyst) did not change because their mass share was always calculated in relation to the current masses of polyol raw materials and polyisocyanate. Although the content of C, H, O, N, was variable in all foams, it was still close to the composition of the reference foam.

**Figure 11:**
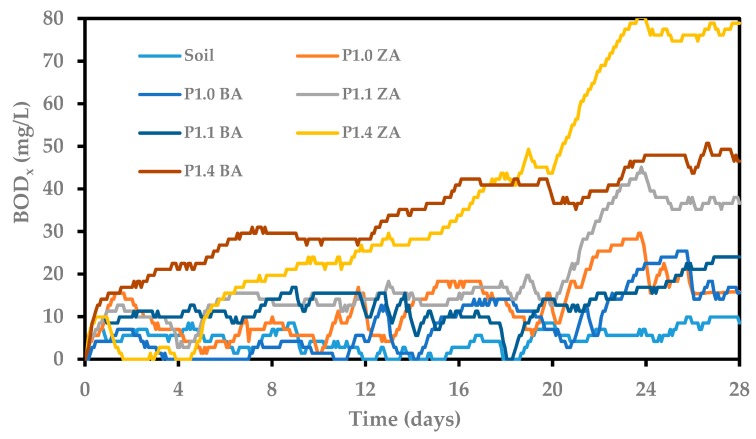
Results of biochemical oxygen demand of RPU/PIR foams during 28 days in soil.

Samples prepared in accordance with ISO 17556:2012 were tested into the OxiTop Control S6 apparatus, where the changes in the biochemical oxygen demand in the system (BOD_x_) were monitored by the respirometric method for 28 days. The results of BOD_x_ changes for the tested RPU/PIR foams are shown in Figure 11. 

The BOD_28_ values were calculated from Equation (10) based on the results of measured changes in biochemical oxygen demand (Figure 11). It is necessary to determine the degree of polymer biodegradation (D_t_ in Equation (11)) after 28 days in soil. Table 7 presents the results of calculated values of BOD_28_, TOD (calculated on the basis of Equation (12) and the results from Table 6) and the degree of biodegradation of RPU/PIR foams based on white mustard seed oil-based bio-polyol.

An increase in biochemical oxygen demand was observed, along with an increase in the content of bio-polyol in RPU/PIR foams in both series. The highest values of this parameter (e.g., 70.4 mg/L) were noted for foams modified with the highest amount of white mustard oil-based bio-polyol. The obtained results of biochemical oxygen demand were several times higher than for reference foams. The degree of polymer biodegradation (D_t_) was determined based on the obtained results. Biodegradation degrees for reference foams were 8.0% (ZA series) and 7.9% (BA series), respectively, whereas, the D_t_ for foams modified by bio-polyol were from 32.4 to 74.1% for the ZA series and from 16.4 to 39.0% for the BA series. The process of biological degradation of RPU/PIR foams was better in foams with a commercial flame retardant than in foams without its participation. An important factor causing the increase of D_t_ in modified foams (both in the ZA and BA series) was the presence of a sulfur heteroatom in the polyurethane structure. This element is a so-called biogenic element, meaning that it is necessary for the proper functioning of all living organisms. The literature provides information that the presence of sulfur in soil causes an increase in the development of microorganisms contained in the soil [68]. Thus, the addition of this element in the structure of a compound of natural origin (vegetable oil) favored its release into the biodegradation environment (garden soil), which led to an increase in the overall biodegradation of these materials.

The high biodegradation degree of the polymer is undoubtedly a big advantage of the obtained RPU/PIR foams. In particular, it is much higher for modified foams than for the reference foams. This suggests that the presence of elements, such as carbon, hydrogen, oxygen, nitrogen, or sulfur, introduced in the plant raw materials, can significantly improve the biodegradation ability of rigid foams. However, the scale of the whole measurement should also be noted. To carry out this process, the RPU/PIR foam samples were mixed with soil in a mass ratio of 1 to 1500 (200 mg of foam to 200 g of dry soil and 100 g of distilled water). This means that proper preparation of biodegradable material and very large excess of matter constituting the biodegradation environment are required [69].

## 4. Conclusions

The use of bio-polyol based on mustard seed oil in the mixture with petrochemical polyol allowed us to obtain new polyurethane materials with properties similar to or better than commercially available ones. The increase in content of plant-oil based polyol caused a slight increase in technological times. It was also found that the use of bio-polyol had positive influence on the properties of rigid polyurethane–polyisocyanurate foams. RPU/PIR foams obtained based on bio-polyol had a lower apparent density (about 35 kg/m^3^), lower water absorption (about 1.5–2%), and lower thermal conductivity coefficient (about 25 mW/(m·K)) than foams based on petrochemical polyol. Furthermore, the obtained materials were more resistant to aging. The use of polyol, which contain sulfur heteroatoms, significantly reduced the flammability of the obtained polyurethane materials. All foams based on PG1 bio-polyol had LOI above 21%, which classified them as flame-retardant materials. The use of bio-polyols and flame retardant in the formulation allowed the introduction of sulfur and phosphorus compounds into the RPU/PIR foam structure. The addition of these compounds caused a flame-retardant effect through the synergistic interaction between them. Chlorine and bromine containing flame retardants can be eliminated due to this phenomenon because they cause environmental controversy. Therefore, their use in some sectors of the economy (e.g., in electrical engineering) is forbidden. It should be noted that such restrictions have not yet been introduced in civil engineering to which this article referred. The greatest advantage of RPU/PIR foams based on white mustard oil-based polyol and 2,2’-thiodiethanol was its biodegradability in soil. The degree of foam biodegradation of the ZA series with the highest content of bio-polyol was 74.1% in 28 days. The main disadvantage of these materials was the decrease of compressive strength. However, its final value was within the range of values assumed for this type of polyurethane materials (above 280 kPa).

The research results presented in this article indicate a very high application potential of bio-polyols based on white mustard seed oil for the production of rigid polyurethane–polyisocyanurate foams dedicated for thermal insulation. The nature-based raw material used in this study is a perfect example of implementation of sustainable development rules in the chemical technology and technology of polymeric materials. The use of renewable materials as a substitute for petrochemical raw materials is currently the leading-edge trend in the polyurethane industry. We can obtain alternative products to conventional materials and take care of the natural environment at the same time.

## 5. Patents

The research results are protected by the Polish Patent No. PL 233221.

## Figures and Tables

**Table 1:** Formulation of rigid polyurethane–polyisocyanurate (RPU/PIR) foams.

Foam Symbol	Rokopol RF-551 (*Eq*) (g)	PG1 (*Eq*) (g)	Tegostab 8460 (g)	33% DABCO (g)	33% Potassium Acetate (g)	Antiblaze TCMP (g)	Solkane HFC 365/227 (g)	Purocyn B (*Eq*) (g)
**P1.0 ZA**	1.0	0.0	4.59	2.70	6.75	45.90	32.40	3.0
	66.80	0.00						203.2
**P1.1 ZA**	0.9	0.1	4.60	2.71	6.77	46.04	32.75	3.0
	60.12	9.59						203.2
**P1.2 ZA**	0.8	0.2	4.62	2.71	6.79	46.19	33.10	3.0
	53.44	19.18						203.2
**P1.3 ZA**	0.7	0.3	4.63	2.72	6.81	46.34	33.45	3.0
	46.76	28.78						203.2
**P1.4 ZA**	0.6	0.4	4.64	2.73	6.84	46.48	33.80	3.0
	40.08	38.37						203.2
**P1.0 BA**	1.0	0.0	4.59	2.70	6.75	0.00	32.40	3.0
	66.80	0.00						203.2
**P1.1 BA**	0.9	0.1	4.60	2.71	6.77	0.00	32.75	3.0
	60.12	9.59						203.2
**P1.2 BA**	0.8	0.2	4.62	2.71	6.79	0.00	33.10	3.0
	53.44	19.18						203.2
**P1.3 BA**	0.7	0.3	4.63	2.72	6.81	0.00	33.45	3.0
	46.76	28.78						203.2
**P1.4 BA**	0.6	0.4	4.64	2.73	6.84	0.00	33.80	3.0
	40.08	38.37						203.2

**Table 2:** Processing times of RPU/PIR foams based on bio-polyol.

Foam Symbol	Cream Time (s)	String Gel Time (s)	Tack-Free Time (s)	Free Rise Time (s)
P1.0 ZA	6	21	24	34
P1.1 ZA	7	22	25	37
P1.2 ZA	7	23	27	44
P1.3 ZA	9	24	29	46
P1.4 ZA	9	31	35	47
P1.0 BA	9	20	23	29
P1.1 BA	9	20	23	29
P1.2 BA	9	20	23	31
P1.3 BA	9	20	23	33
P1.4 BA	9	20	23	34

**Table 3:** Physico-mechanical properties comparison of commercial RPU/PIR foam for thermal insulation and foams with 0.4 *Eq* of bio-polyol content.

Parameter	Apparent Density (kg/m^3^)	Compressive Strength (kPa)	Water Absorption after 24 h (%)
G40 *	36.0 - 42.0	> 250.00	< 3.00
P1.4 ZA	38.4 ± 1.1	289.95 ± 7.46	1.81 ± 0.06
P1.4 BA	35.9 ± 1.3	303.67 ± 5.32	0.96 ± 0.03

* commercial RPU/PIR foam [37].

**Table 4:** Results of SEM micrographs analysis.

Foam Symbol	Cell Size (μm)	Thickness of Cell Wall (μm)	Content of Cells per area unit (cells/mm^2^)
P1.0 ZA	248 ± 26	18 ± 2	15 ± 3
P1.1 ZA	263 ± 31	22 ± 2	13 ± 3
P1.4 ZA	296 ± 41	27 ± 3	11 ± 4
P1.0 BA	273 ± 34	19 ± 2	13 ± 2
P1.1 BA	294 ± 29	22 ± 3	12 ± 4
P1.4 BA	317 ± 43	27 ± 2	11 ± 4

**Table 5:** Fire properties of selected RPU/PIR foams.

Foam Symbol	TTI (s)	TTF (s)	HRR (kW/m^2^)	THR (MJ/m^2^)
P1.0 ZA	10 ± 1	128 ± 2	78.9 ± 0.8	16.9 ± 0.5
P1.4 ZA	21 ± 1	69 ± 2	114.8 ± 1.3	9.7 ± 0.3
P1.0 BA	6 ± 1	175 ± 3	69.4 ± 0.7	23.1 ± 0.9
P1.4 BA	12 ± 1	125 ± 1	86.7 ± 0.7	15.6 ± 0.3

**Table 6:** Calculated mass shares of individual elements.

Element	C	H	O	Si	N	S	P	Cl	K	F
**PG1.0 ZA**	0.599	0.057	0.168	0.005	0.059	0.000	0.012	0.041	0.002	0.058
**PG1.1 ZA**	0.6	0.059	0.164	0.005	0.058	0.002	0.012	0.041	0.002	0.058
**PG1.4 ZA**	0.603	0.063	0.153	0.005	0.056	0.007	0.012	0.041	0.002	0.058
**PG1.0 BA**	0.638	0.057	0.164	0.005	0.067	0.000	0.000	0.000	0.003	0.066
**PG1.1 BA**	0.639	0.059	0.159	0.005	0.066	0.002	0.000	0.000	0.003	0.066
**PG1.4 BA**	0.643	0.065	0.146	0.005	0.064	0.008	0.000	0.000	0.003	0.066

**Table 7:** Results of biodegradability RPU/PIR foams.

Foam symbol	PG1.0 ZA	PG1.1 ZA	PG1.4 ZA	PG1.0 BA	PG1.1 BA	PG1.4 BA
**BOD_28_ (mg/L)**	7.0	28.1	70.4	7.1	15.5	38.0
**TOD (mg/L)**	87.2	86.7	95.0	90.3	94.6	97.5
**D_t_ (%)**	8.0	32.4	74.1	7.9	16.4	39.0

## Supplementary Materials

The following are available online at https://www.mdpi.com/2073-4360/11/11/1816/s1.
