# Supplementary material for: Biodegradable, Flame-Retardant, and Bio-Based Rigid Polyurethane/Polyisocyanurate Foams for Thermal Insulation Application
Source: Polymers (Basel). 2019 Nov 5;11(11):1816. doi: 10.3390/polym11111816 (PMC6918136; doi:10.3390/polym11111816)
Supplement: Supplementary file 1 [file polymers-11-01816-s001.pdf]

Biodegradable, flame-retardant and bio-based rigid polyurethane/polyisocyanurate foams for thermal insulation application

Marcin Borowicz [1,*], Joanna Paciorek-Sadowska [1,*], Jacek Lubczak [2] and Bogusław Czupryński [1]

[1] Department of Chemistry and Technology of Polyurethanes, Institute of Materials Engineering, Kazimierz Wielki University, J. K. Chodkiewicza Street 30, 85-064 Bydgoszcz, Poland; insttech@ukw.edu.pl

[2] Department of Organic Chemistry, Rzeszów University of Technology, Al. Powstańców Warszawy 6, 35-959 Rzeszów, Poland. jml@prz.edu.pl

* Correspondence: m.borowicz@ukw.edu.pl; Tel.: +48 34 19 286; sadowska@ukw.edu.pl; Tel.: +48 34 19 289;

1. Nuclear magnetic resonance (NMR) spectroscopy analysis of PG1 bio-polyol

The chemical structure of oil, epoxidized oil and bio-polyol was analyzed by ^{1}H NMR and ^{13}C NMR spectroscopy at 400 MHz using an Ascend III NMR spectrometer (Brücker, Billerica, MA, USA). Deuterated chloroform was used as a solvent for the above mentioned samples.

1.1. ^{1}H NMR analysis

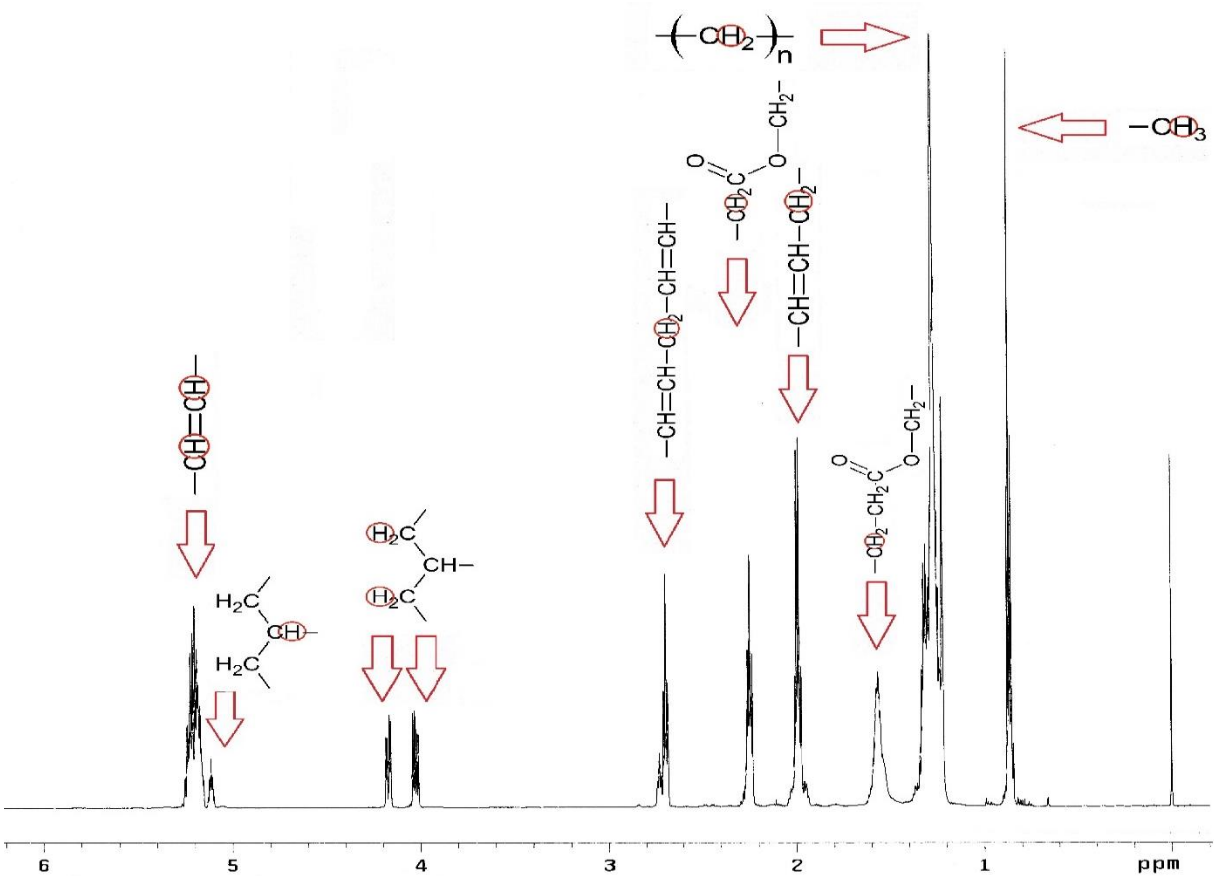

Figure S1. ^{1}H NMR spectra of mustard oil.

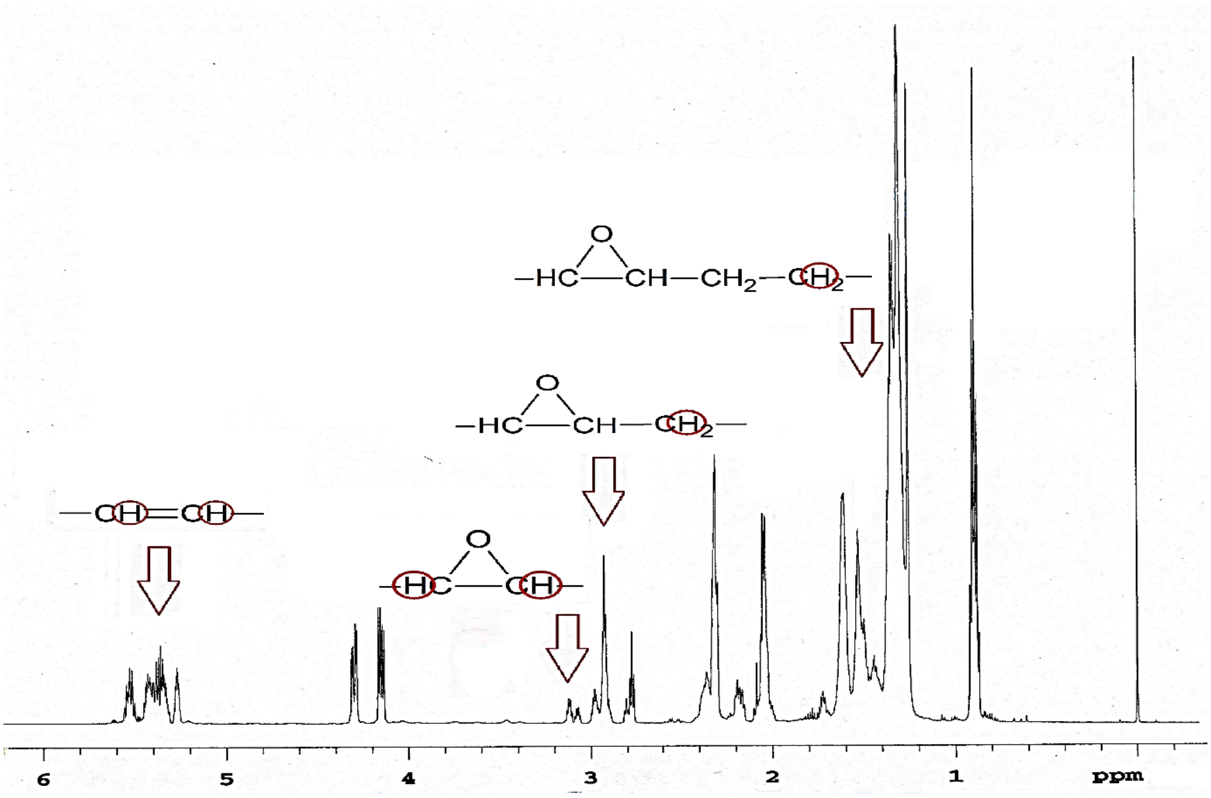

Figure S2. ^{1}H NMR spectra of epoxidized mustard oil.

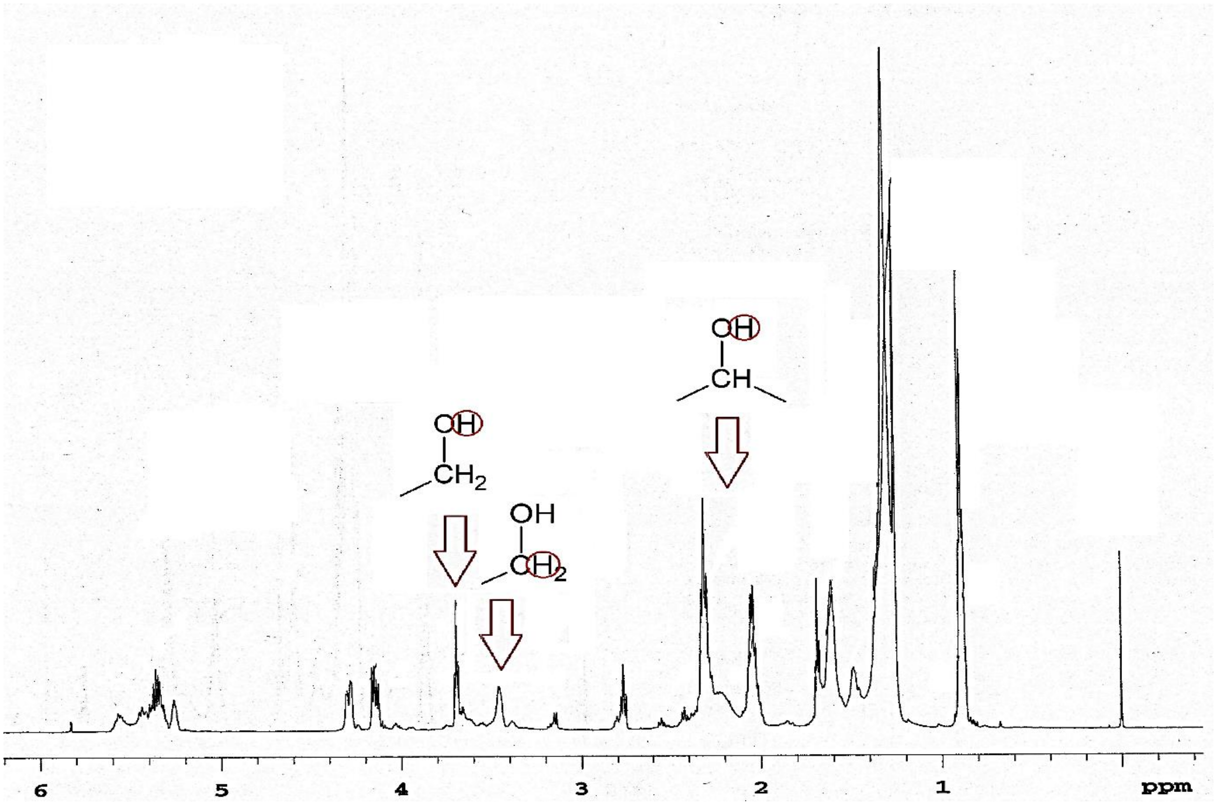

Figure S3. ^{1}H NMR spectra of mustard oil-based bio-polyol PG1.

Analysis of ^{1}H NMR spectra of white mustard seed oil (Figure S1) showed the presence of characteristic chemical shifts for: 5.35 ppm – protons of olefin groups –CH=CH–; 5.25 ppm – methane protons of glyceryl $–CH_2–CH–CH_2–$; 4.12-4.28 ppm – methylene protons of glyceryl $–CH_2–CH–CH_2–$; 2.70-2.80 ppm – protons of bis-allyl methylene groups $–CH=CH–CH_2–CH=CH–$; 2.29-2.32 ppm – protons of α-CH_2 groups in relation to the carbonyl (ester) group $–CH_2–CO–$; 2.10-2.25 ppm – protons

of α-CH_2 groups in relation to the olefin groups –CH_2–C**H**$_2$–CH=CH–; 1.60 ppm – protons of β-CH_2 groups in relation to the carbonyl (ester) group –C**H**$_2$–CH_2–CO–; 1.25-1.40 ppm – protons of –C**H**$_2$– groups in fatty acid chains; 0.86-0.88 ppm – protons of –C**H**$_3$ end groups.

The ^{1}H NMR spectrum of epoxidized mustard oil and bio-polyol based on it (Figure S2 and S3) showed the presence of characteristic chemical shifts which also occurred in white mustard seed oil (Figure S1). Only in the spectrum of epoxidized oil (Figure S2) characteristic chemical shifts for the epoxy group were observed: 3.17-3.19 ppm – protons of epoxy groups –C**H**–(O)–C**H**–; 2.85-2.96 ppm – protons of α-CH_2 groups in relation to the epoxy group –CH–(O)–CH–C**H**$_2$– and 1.45-1.50 ppm – protons of β-CH_2 groups in relation to the epoxy group –CH–(O)–CH–CH_2–C**H**$_2$–. In the spectrum of bio-polyol based on mustard oil (Figure S3), disappearance of characteristic shifts for epoxy groups and the presence of new shifts were noted in: 3.70-3.80 ppm – protons of hydroxyl groups at the end of the chain –O**H**; 3.42-3.50 ppm – protons of α-CH_2 groups in relation to the hydroxyl group –C**H**$_2$–OH and 2.15-2.25 ppm – protons of hydroxyl groups inside the chain >CH–O**H**.

1.2. ^{13}C NMR analysis

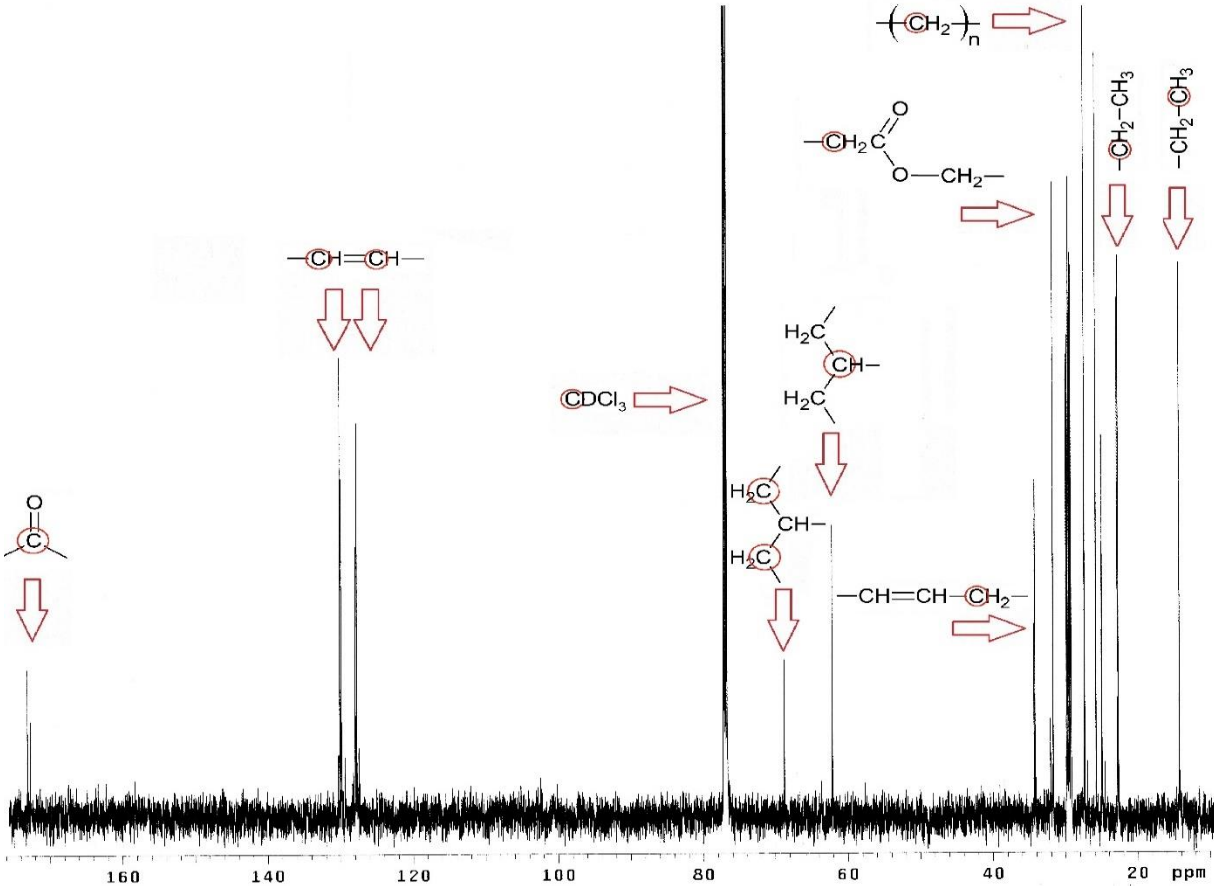

Figure S4. ^{13}C NMR spectra of mustard oil.

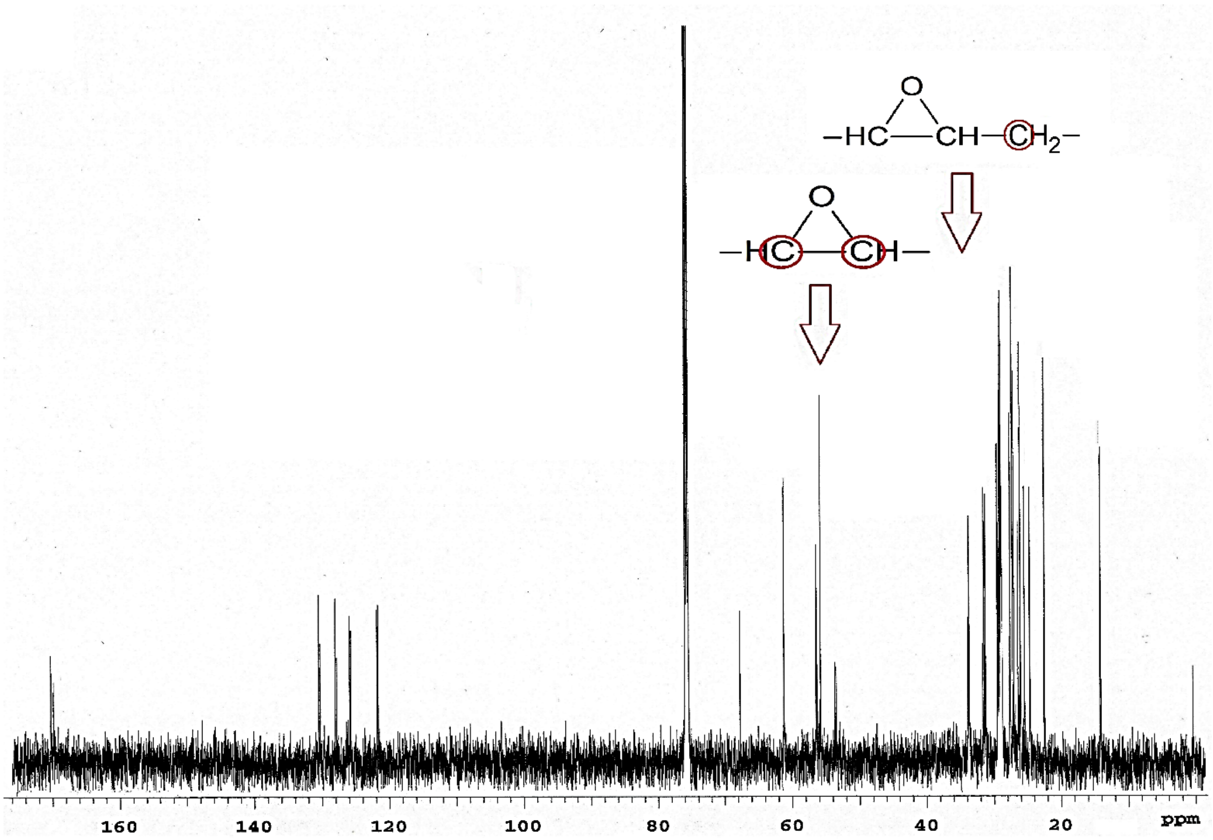

Figure S5. ^{13}C NMR spectra of epoxidized mustard oil.

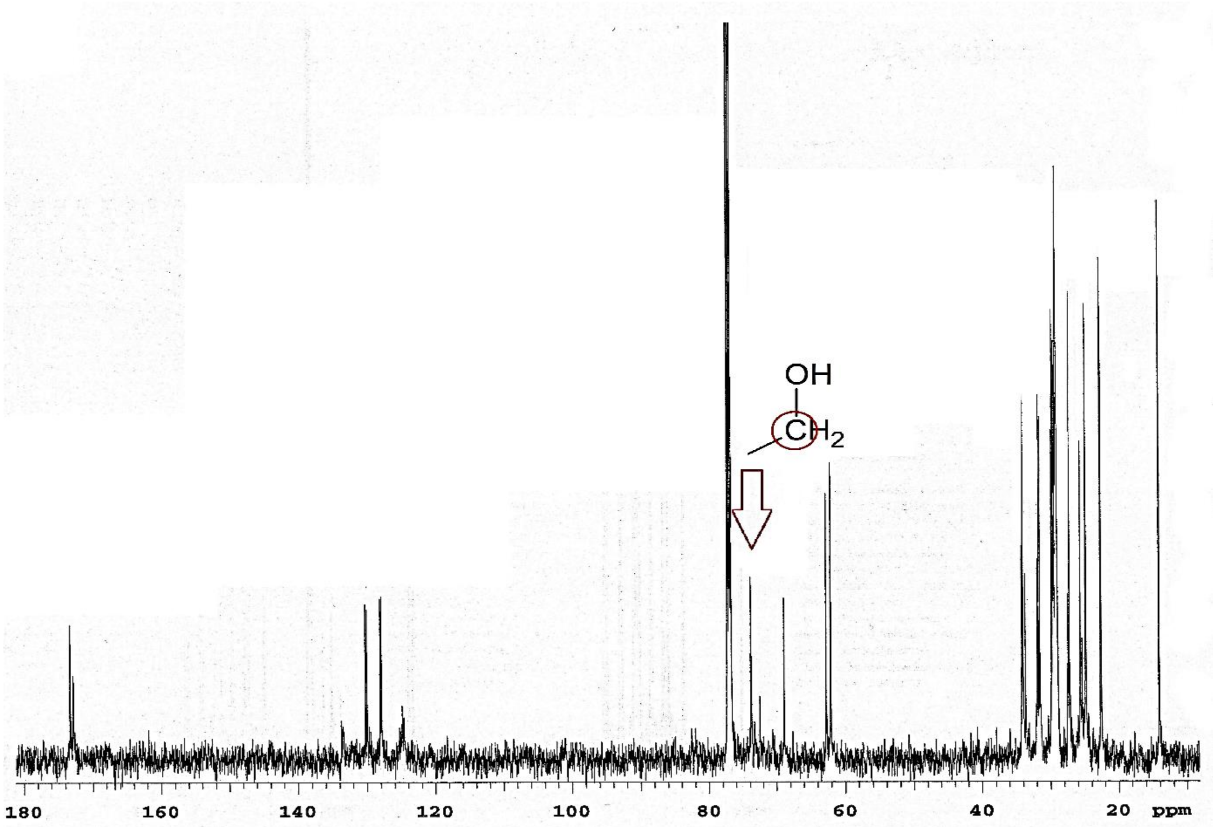

Figure S6. ^{13}C NMR spectra of mustard oil-based bio-polyol PG1.

Analysis of ^{13}C NMR spectrum of white mustard seed oil (Figure S4) showed the presence of characteristic chemical shifts for: 172.80-173.25 ppm – carbons of carbonyl groups >C=O; 127.10-132.30

ppm – carbons of olefin group in fatty acids –CH=CH–; 68.90 ppm – methane carbons in glyceryl –CH_2–CH–CH_2–; 62.10 ppm – methylene carbons in glyceryl –CH_2–CH–CH_2–; 33.00 ppm – carbons of α-CH_2 groups in relation to the olefin groups –CH=CH–CH_2–; 31.90 ppm – carbons of α-CH_2 groups in relation to the carbonyl groups –CH_2–OOC–CH_2-; 27.20-29.80 ppm – carbons of CH_2 groups in fatty acid chains; 22.70 ppm – carbons of the penultimate CH_2 groups in the fatty acid chain –CH_2–CH_3; 14.30 ppm – carbons of end groups –CH_3.

The analysis of ^{13}C NMR spectrum of epoxidized mustard oil and bio-polyol based on it (Figures S5 and S6) showed the presence of characteristic chemical shifts which also occurred in white mustard seed oil (Figure S4). Additional chemical shifts were observed in the spectrum of epoxidized oil (Figure S5) in: 57.20 ppm – carbons of the epoxy group –CH–(O)–CH– i 34.00 ppm – carbons of α-CH_2 groups in relation to the epoxy group –CH_2–CH–(O)–CH–. While, on the bio-polyol spectrum (Figure S6), additional chemical shifts were noted in: 73.86 ppm – carbons of α-CH_2 groups in relation to the hydroxyl groups at the end of the chain –CH_2–OH. In addition, peaks characteristic of epoxy groups have disappeared.
